# Marginal bone loss around dental implants: comparison between matched groups of bruxer and non‐bruxer patients: A retrospective case–control study

**DOI:** 10.1111/cid.13161

**Published:** 2022-11-21

**Authors:** Clara Bredberg, Camila Vu, Birgitta Häggman‐Henrikson, Bruno Ramos Chrcanovic

**Affiliations:** ^1^ Faculty of Odontology Malmö University Malmö Sweden; ^2^ Department of Orofacial Pain and Jaw Function, Faculty of Odontology Malmö University Malmö Sweden; ^3^ Department of Prosthodontics, Faculty of Odontology Malmö University Malmö Sweden

**Keywords:** bruxism, dental implants, marginal bone loss, retrospective clinical study

## Abstract

**Purpose:**

To compare marginal bone loss (MBL) around dental implants in a group of bruxers in relation to a matched group of non‐bruxers.

**Methods:**

The present record‐based retrospective study included patients selected from individuals treated with dental implants at one specialist clinic in Malmö. Only implants not lost and with baseline radiographs taken within 12 months after implant placement and with a minimum of 36 months of radiological follow‐up were considered for inclusion. Univariate linear regression models and a linear mixed‐effects model were performed.

**Results:**

Two hundred and four patients (104 bruxers, 100 non‐bruxers), with a total of 811 implants (416 in bruxers, 395 in non‐bruxers) were included in the study. The results of the linear mixed‐effects model suggested that bruxism, smoking, age, region of the jaws, implant diameter, and prosthesis type had a statistically significant influence on MBL over time. Individuals who are both bruxers and smokers showed greater MBL when compared to individuals who are either a bruxer or smoker, or neither (*p* < 0.001).

**Conclusions:**

Bruxism is suggested to increase the risk of MBL over time, as well as higher age, smoking, and the combination of bruxism and smoking. Other factors that showed a correlation with increased MBL were implant diameter, region of the jaws, and prosthesis type, but it is not possible to draw robust conclusions for these factors, as the categories of these variables were very unbalanced.

**What is known**

Some studies have suggested that mechanical stresses exceeding the biological load‐bearing capacity of a dental implant could be associated with marginal bone loss (MBL), but clinical studies comparing MBL between bruxers and non‐bruxers are still lacking.

**What this study adds**

This study is the first one to compare marginal bone loss around implants in matched groups of bruxer and non‐bruxer patients. The results suggest that bruxism negatively affects the marginal bone level around implants over time. The combination of bruxism and smoking results in an even worse outcome.

## 1 INTRODUCTION

Bruxism is generally defined as excessive grinding of teeth or jaw clenching, during sleep or wakefulness, and is considered as a parafunctional activity. The condition has been reported to occur in about 8% of adults, although some claim that it is even more prevalent, occurring in 31.4% of adults.^1^ The importance of the condition is based on the fact that bruxism has been associated with many negative consequences, such as excessive mechanical tooth wear,^2^ prosthodontic technical complications,^3^, ^4^ temporomandibular disorders,^5^ headache,^6^, ^7^ and orofacial pain,^8^ among others.

Regarding dental implants, clinical studies suggest that bruxism may be associated with higher prevalence of technical complications and failures of implant‐supported prostheses,^9^, ^10^, ^11^, ^12^, ^13^, ^14^, ^15^ higher implant failure rate,^16^, ^17^ and even an increased risk of implant fracture.^18^ Regarding marginal bone loss (MBL) around implants, the literature is scarce when it comes to bruxism. Some studies^19^, ^20^, ^21^ have suggested that mechanical stresses exceeding the biological load‐bearing capacity of a dental implant could be associated with MBL. Some animal studies have suggested that excessive occlusal load on dental implants does not cause significant changes in their radiological outcomes,^22^, ^23^ whereas another study suggested that bone resorption around implants could be caused by excess occlusal trauma, even when there is no inflammation in the peri‐implant tissue.^24^ The results of another animal experimental study suggest that excessive dynamic loads may cause crater‐like bone defects lateral to osseointegrated implants, in comparison to static‐loaded implants.^25^ Additionally, it was suggested that excessive lateral static load may negatively affect the behavior of peri‐implant bone around immediately restored implants.^26^ However, besides the excessive occlusal load, these animal studies did not really simulate the complexity of the parafunctional activity resulting from bruxism. Furthermore, clinical studies comparing MBL between bruxers and non‐bruxers in humans are still lacking.

Therefore, it was the aim of the present retrospective study to analyze the MBL around dental implants in a group of patients presenting bruxism in comparison with a matched group of non‐bruxers.

## 2 MATERIALS AND METHODS

### 2.1 Research question and hypothesis

The focused question was elaborated by using the PICO format (Participants, Interventions, Comparisons, Outcomes): “Do bruxers undergoing implant‐prosthetic rehabilitation present a higher MBL over time in comparison to patients not presenting bruxism?”

The null hypothesis was that there would be no difference in MBL between bruxers and non‐bruxers, against the alternative hypothesis of a difference.

### 2.2 Patients

This retrospective study included patients treated by certified oral surgeons and prosthodontists with dental implants and implant‐supported prostheses during the period 1980–2018 at one specialist clinic (Clinic for Prosthodontics, Centre of Dental Specialist Care, Malmö, Sweden). This study was based on data collection from patients' dental records.

The study was approved by the regional Ethical Committee, Lund, Sweden (Dnr 2014/598; Dnr 2015/72), and was registered at the Registry of Clinical Trials (https://clinicaltrials.gov) under the registration number NCT02369562.

### 2.3 Definitions

MBL was defined as loss, in an apical direction, of alveolar bone marginally adjacent to the dental implant, in relation to the marginal bone level initially detected after the implant was surgically placed.

For this study, the authors followed the definition of bruxism proposed by Lobbezoo and colleagues^27^: “*repetitive masticatory muscle activity characterized by clenching or grinding of the teeth and/or by bracing or thrusting of the mandible and specified as either sleep bruxism or awake bruxism*”. The signs and symptoms of bruxism were listed according to the International Classification of Sleep Disorders,^28^ following the same guidelines used in a recent study.^12^ The patients suspected to be bruxers, as diagnosed in the records, were recalled in this previous study in order to be clinically re‐assessed. The clinical examination included evaluation of the presence of masticatory muscle hypertrophy, indentations on the tongue or lip and/or a *linea alba* on the inner cheek, damage to the dental hard tissues (e.g., cracked teeth), repetitive failures of restorative work/prosthodontic constructions, or mechanical wear of the teeth (i.e., attrition).^27^ Moreover, the self‐conscience of the condition was evaluated with five questions, according to suggestions from a previous study.^29^ A diagnostic grading system of ‘possible’, ‘probable’ and ‘definite’ sleep or awake bruxism was used, as suggested for clinical and research purposes.^30^ The patients from a present study^12^ were eligible for the present study and classified as ‘probable’ sleep or awake bruxers, based on anamnesis/self‐report together with the clinical examination.

### 2.4 Inclusion and exclusion criteria

All patients diagnosed as ‘probable bruxers’ were included. Only implants in situ and with baseline radiographs taken within 12 months after implant placement and with a minimum of 36 months of radiological follow‐up were considered for inclusion. Negative values of MBL corresponded to bone loss.

Only modern types of threaded implants with cylindrical or conical designs were included. Zygomatic implants and implants detected in radiographs, but without basic information registered in the patients' records, were not included in the study.

Patients were excluded if they presented a recent history of periodontitis. It is important to take note that as standard, all patients receiving implants at the Specialist Clinic for Prosthodontics were periodontally healthy at the time of implant installation.

### 2.5 Data collection

The data were directly entered into an SPSS file (SPSS software, version 28, SPSS Inc., Chicago, Illinois) as the records of the patients were being read, and it consisted of the following variables: patient age at implant installation, patient sex, probable bruxism (yes/no), smoking habit (yes/no), number of cigarettes/day, implant location (jaw and tooth region), implant diameter (three groups: 3.00–3.50, 3.75–4.10, and 4.30–5.00 mm), implant surface (turned/machined, modified), prosthesis type (single crown, fixed dental prosthesis (FDP) with 2 to 6 prosthetic units, FDP with 7 to 10 units, full‐arch prosthesis, overdenture), prosthesis fixation type (cemented, screwed), prosthesis material (metal acrylic, metal ceramic, full ceramic, zirconia, acrylic).

### 2.6 Formation of a matched group

Since the division of all initial patients into groups would generate extremely unbalanced groups and the variance was not homogenous between them, the two groups (bruxers and non‐bruxers) were not expected to be comparable with respect to important covariates,^31^ and methods to match patients and implants between bruxer to non‐bruxer patients were used. Matching ensures that any differences between the study and the control groups are not a result of differences in the matching variables, thus reducing selection bias.

The matching was performed using the ‘case–control matching’ function in SPSS, and the matches were selected based on (a) patient sex, (b) patient age at the time of the surgery, (c) number of implants, and (d) total radiological follow‐up time. As there were no perfect matches in a first matching attempt for all four variables, some tolerance was set for three of the predictors: ±7 years for the age of the patient, ±12 months for the total radiological follow‐up time, and ±2 for the number of implants. Thus, some variance of these predictors between the groups was expected. Moreover, as the matching was performed at the patient‐level, and as there was some tolerance for the number of implants in the matching, some variance of the variables at the implant‐level between the groups (bruxers, non‐bruxers) was also expected.

### 2.7 Marginal bone level evaluation

Only periapical radiographs were considered for the study. The process of periapical radiograph acquisition at the clinic is standardized, using the long cone paralleling technique. When there were no available digital radiographs from the baseline appointment, analogue periapical radiographs were scanned at 1200 dpi (Epson Perfection V800 Photo Color Scanner; Nagano, Japan). Marginal bone level was measured after calibration, based on the inter‐thread distance of the implants. Measurements were taken from the implant‐abutment junction to the marginal bone level, at both mesial and distal sides of each implant, and then the mean value of these two measurements was considered. MBL was calculated by comparing subsequent bone‐to‐implant contact levels to the radiographic baseline examination. The Image J software (National Institute of Health, Bethesda) was used for all measurements.

The sets of radiographs for every patient were coded and the authors who performed the radiological measurements (C.V., C.B.) were blinded to the diagnosis of the condition for every patient.

### 2.8 Calibration

An initial calibration concerning marginal bone level was performed between the authors. The process was done for 10 random samples from the cohort group, and verified after the measurement of each sample. At the end of the process, the measurements from the different individuals were considered enough approximate from each other, with an agreement between examiners set at >80% of the distance in millimeters.

### 2.9 Sample size calculation

Calculation of the sample size was not conducted. The reason was that the database from which the eligible cases for the present study were originated had a certain number of patients and dental implants, approximately 2800 and 11 000 respectively, and it would not possible to recruit more cases, as the database already included all patients treated with dental implants during the aforementioned period at the specialist clinic.

Instead, all the bruxer patients were initially considered eligible for inclusion, in order to get the maximum number of cases available, namely, the largest sample size possible from this database, provided that these cases would fulfill the inclusion criteria, that is, baseline radiographs taken within 12 months after implant placement and with a minimum of 36 months of radiological follow‐up. The number of implants in the bruxer patients was then matched to a group of non‐bruxer patients.

### 2.10 Statistical analyses

The mean, standard deviation, and percentages were presented as descriptive statistics. Kolmogorov–Smirnov test was performed to evaluate the normal distribution of the variables, and Levene's test evaluated homoscedasticity. The performed tests for two independent groups were Student's *t*‐test or Mann–Whitney test, and for three or more independent groups were ANOVA or Kruskal‐Wallis test, depending on the normality. Paired *t*‐test or Wilcoxon Signed Rank test was used to compare the mean value difference of continuous variables between dependent groups. Pearson's chi‐squared test or Fisher's exact test was used in the analysis of contingency tables of categorical data of independent groups, and McNemar's test was used for dependent groups.

Univariate linear regression models were used to compare MBL over time between the clinical covariates. In order to verify multicollinearity, a correlation matrix of all predictor variables was scanned, to verify whether there were some high correlations among the predictors. Collinearity statistics obtaining variance inflation factor (VIF) and tolerance statistic were also performed to detect more subtle forms of multicollinearity. A linear mixed‐effects model was built with all variables that were moderately associated (*p* < 0.10) with MBL in the univariate linear regression models. Mixed‐effects model was used in order to take into consideration that some patients had more than one implant‐supported prosthesis, as multiple observations within an individual are not independent of each other.

The degree of statistical significance was considered *p* < 0.05. Data were statistically analyzed using the Statistical Package for the Social Sciences (SPSS) version 28 software (SPSS Inc., Chicago, Illinois).

The present retrospective study followed the STROBE guidelines for observational studies.

## 3 RESULTS

There were 106 patients diagnosed as probable bruxers in the cohort group, with baseline radiographs taken within 12 months after implant placement and radiographs with a minimum of 36 months of radiological follow‐up. These 106 bruxers were matched to 106 patients known not to be bruxers, and fulfilling the same radiological inclusion criteria. The periapical radiographs for two bruxers (1.9% of this group) and six non‐bruxer (5.7%) patients were excluded for not being of sufficient quality. Therefore, 204 patients were included in the present study (104 bruxers, 100 non‐bruxers), with 811 dental implants (416 in bruxers, 395 in non‐bruxers).

The mean age (±SD) of the 204 patients was 56.1 ± 14.5 years (min–max, 16.9–89.1) on the day of implant placement. The patients were followed up clinically for a mean (±SD) of 159.4 ± 81.8 months (min–max, 38.7–381.8), and radiographically for a mean (±SD) of 127.4 ± 76.3 months (min–max, 36.4–363.0).

Table 1 shows the descriptive data of the cases included in the study, separated by group. The variable of patient's age was divided into three categories each, based on the 33.3 and 66.7 percentiles of sample distribution, in order to generate groups of more balanced sample sizes.

**TABLE 1:** Descriptive data of the implants included in the study, separated by group. The statistical unit is the implant, not the patient.

Factor	Bruxers Implants (%)	Non‐bruxers Implants (%)	*p* value
*Follow‐up* (*months*)			
(mean ± SD)	165.5 ± 82.8	153.1 ± 80.3	0.079^a^
*Age*			
(mean ± SD)	55.1 ± 15.1	57.1 ± 13.8	0.216^a^
*Age* (*years*)			
≤52	145 (34.9)	122 (30.9)	**0.001** ^b^
52.1–63.9	152 (36.5)	111 (28.1)	
≥64	119 (28.6)	162 (41.0)	
*Sex*			
Male	234 (56.2)	247 (62.5)	0.069^b^
Female	182 (43.8)	148 (37.5)	
*Jaw*			
Maxilla	265 (63.7)	225 (57.0)	0.050^b^
Mandible	151 (36.3)	170 (43.0)	
*Region*			
Incisive	118 (28.4)	141 (35.7)	**0.027** ^b^
Canine	74 (17.8)	81 (20.5)	
Premolar	177 (42.5)	143 (36.2)	
Molar	47 (11.3)	30 (7.6)	
*Implant surface*			
Turned	227 (54.6)	201 (50.9)	0.294^b^
Modified	189 (45.4)	194 (49.1)	
*Implant diameter*			
3.00–3.50 mm	34 (8.2)	16 (4.1)	**0.033** ^b^
3.75–4.10 mm	371 (89.2)	371 (94.2)	
4.30–5.00 mm	11 (2.6)	7 (1.8)	
*Prosthesis type*			
Single crown	59 (12.1)	49 (12.4)	**0.003**
FDP 2–6 units	160 (38.7)	110 (27.8)	
FDP 7–10 units	13 (3.2)	26 (6.6)	
Full‐arch	185 (44.8)	208 (52.7)	
Overdenture	5 (1.2)	2 (0.5)	
*Prosthesis fixation* ^c^			
Cemented	39 (9.5)	32 (8.2)	0.515^b^
Screwed	373 (90.5)	360 (91.8)	
*Prosthesis material* ^c^			
Metal acrylic	189 (45.9)	223 (57.2)	**0.014** ^b^
Metal ceramic	188 (45.6)	135 (34.6)	
Full ceramic	15 (3.6)	17 (4.4)	
Zirconia	15 (3.6)	13 (3.3)	
Acrylic	5 (1.2)	2 (0.5)	
*Smoking* ^c^			
No	223 (64.3)	277 (72.1)	**0.022** ^b^
Yes^d^	124 (35.7)	107 (27.9)	

Abbreviations: FDP, fixed dental prosthesis; SD, standard deviation.

^a^ Wilcoxon Signed Rank test.

^b^ Comparison of the distribution of cases, among the categories of each factor, between bruxers and non‐bruxers.

^c^ For the cases with available information.

^d^ It includes 48 implants in 9 former smokers.

**FIGURE 1:**
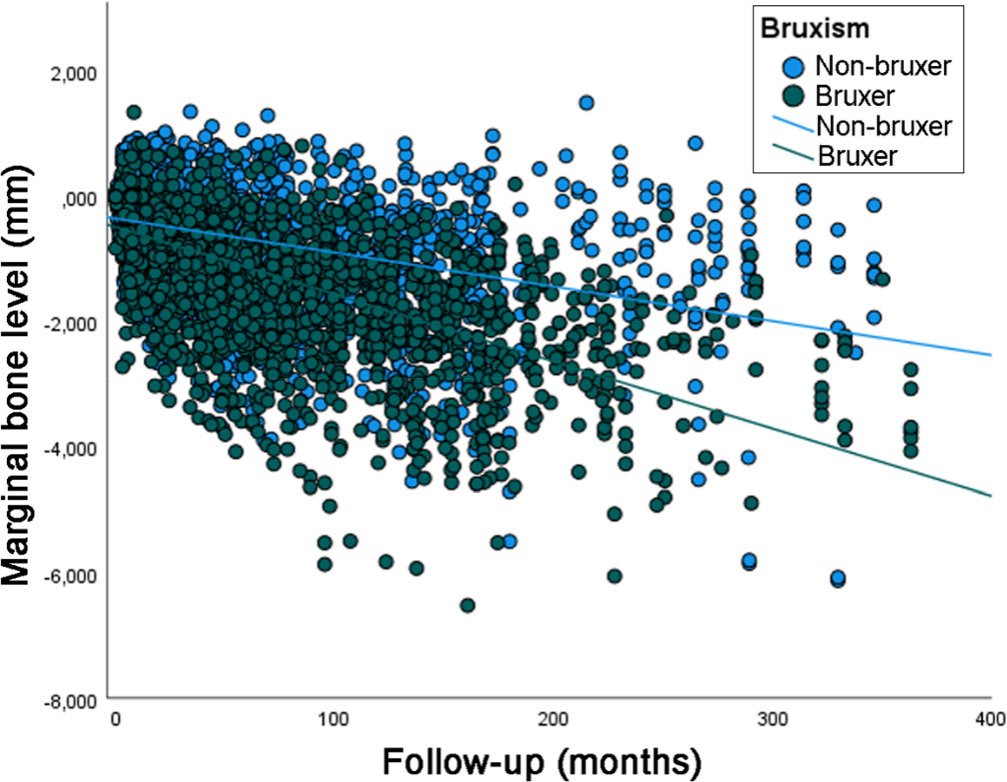
Scatter plot comparing the marginal bone level over time between implants placed in bruxers and non‐bruxer patients (linear regression).

**FIGURE 2:**
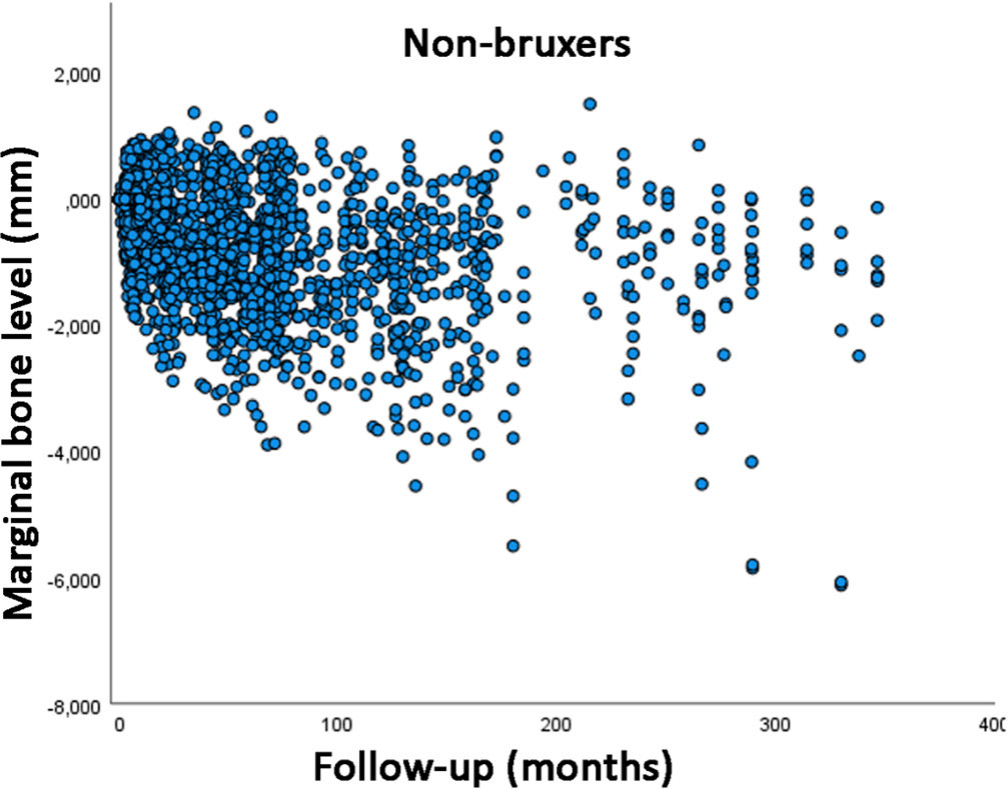
Scatter plot of the marginal bone level measurements in function of time of follow‐up, for non‐bruxer patients.

**FIGURE 3:**
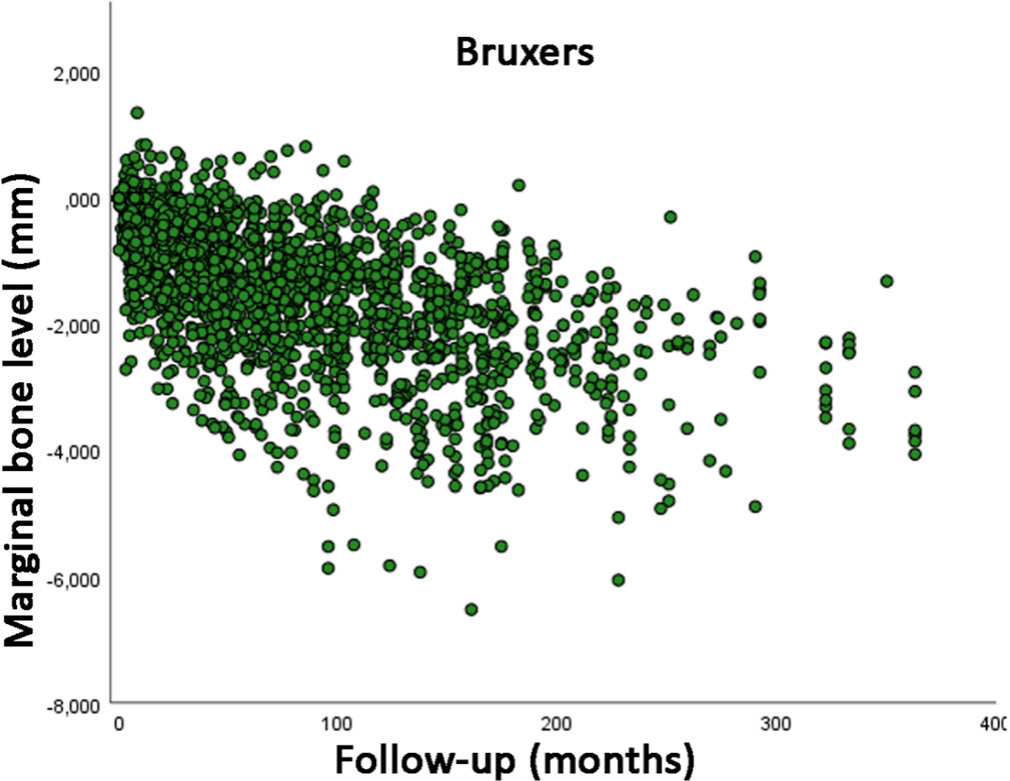
Scatter plot of the marginal bone level measurements in function of time of follow‐up, for probable bruxer patients.

The total number of marginal bone level double measurements (mesial and distal sides of each implant) was 4823, with 2569 double measurements in bruxers and 2254 in non‐bruxers. The univariate linear regression analysis showed that the mean loss of marginal bone over time was statistically significantly different between the categories of all variables (Table 2). The scatter plot with a comparison of MBL over time between bruxers and non‐bruxers is presented (Figure 1). As the superimposition of dots in the scatter plot can give the false impression that most implants in non‐bruxers presented only good results, scatter plots with data separated for non‐bruxers (Figure 2) and for bruxers (Figure 3) are also presented.

**TABLE 2:** Univariate linear regression analysis for MBL

Factor	Linear equation^a^	*p* value^b^	*R* ^2^ linear
*Bruxism*			
No	*y* = −0.35 − 0.00546*x*	**<0.001**	0.146
Yes	*y* = −0.49 − 0.01000*x*		0.417
*Smoking* ^c^			
No	*y* = −0.38 − 0.00795*x*	**<0.001**	0.251
Yes^d^	*y* = −0.54 − 0.00889*x*		0.314
*Age* (*years*)			
≤52	*y* = −0.49 − 0.00769*x*	**<0.001**	0.322
52.1–63.9	*y* = −0.37 − 0.00924*x*		0.280
≥64	*y* = −0.37 − 0.00972*x*		0.239
*Sex*			
Male	*y* = −0.45 − 0.00804*x*	**<0.001**	0.245
Female	*y* = −0.39 − 0.00943*x*		0.353
*Jaw*			
Maxilla	*y* = −0.45 − 0.00762*x*	**<0.001**	0.223
Mandible	*y* = −0.37 − 0.00997*x*		0.389
*Region*			
Incisive	*y* = −0.40 − 0.00922*x*	**<0.001**	0.319
Canine	*y* = −0.39 − 0.00777*x*		0.238
Premolar	*y* = −0.43 − 0.00836*x*		0.269
Molar	*y* = −0.45 − 0.00934*x*		0.361
*Implant surface*			
Turned	*y* = −0.42 − 0.00773*x*	**<0.001**	0.296
Modified	*y* = −0.31 − 0.01000*x*		0.326
*Implant diameter*			
3.00–3.50 mm	*y* = −0.32 − 0.01000*x*	**<0.001**	0.243
3.75–4.10 mm	*y* = −0.41 − 0.00850*x*		0.299
4.30–5.00 mm	*y* = −0.40 − 0.02000*x*		0.497
*Prosthesis type*			
Single crown	*y* = −0.39 − 0.01000*x*	**<0.001**	0.302
FDP 2–6 units	*y* = −0.42 − 0.00952*x*		0.324
FDP 7–10 units	*y* = −0.33 − 0.00874*x*		0.354
Full‐arch	*y* = −0.40 − 0.00778*x*		0.260
Overdenture	*y* = −0.34 − 0.01000*x*		0.789
*Prosthesis fixation* ^c^			
Cemented	*y* = −0.32 − 0.01000*x*	**<0.001**	0.406
Screwed	*y* = −0.42 − 0.00841*x*		0.279

^a^ For the linear equation, “*x*” represents the number of months.

^b^ Comparison of the slope of the equation (variation of MBL in mm in time) between groups.

^c^ For the cases with available information.

^d^ It includes 48 implants in 9 former smokers.

Most categories had a weak degree of linear correlation (*R* ^2^ linear) with MBL over time (Table 2). Some categories presented a moderate degree of linear correlation, namely, bruxers, women, implants in mandibles, implants in the molar region, implants with diameter 4.30–5.00 mm, implants supporting prostheses with 7–10 units, and implants with cemented prosthesis. One category presented a strong degree of linear correlation, namely, implants supporting an overdenture.

**FIGURE 4:**
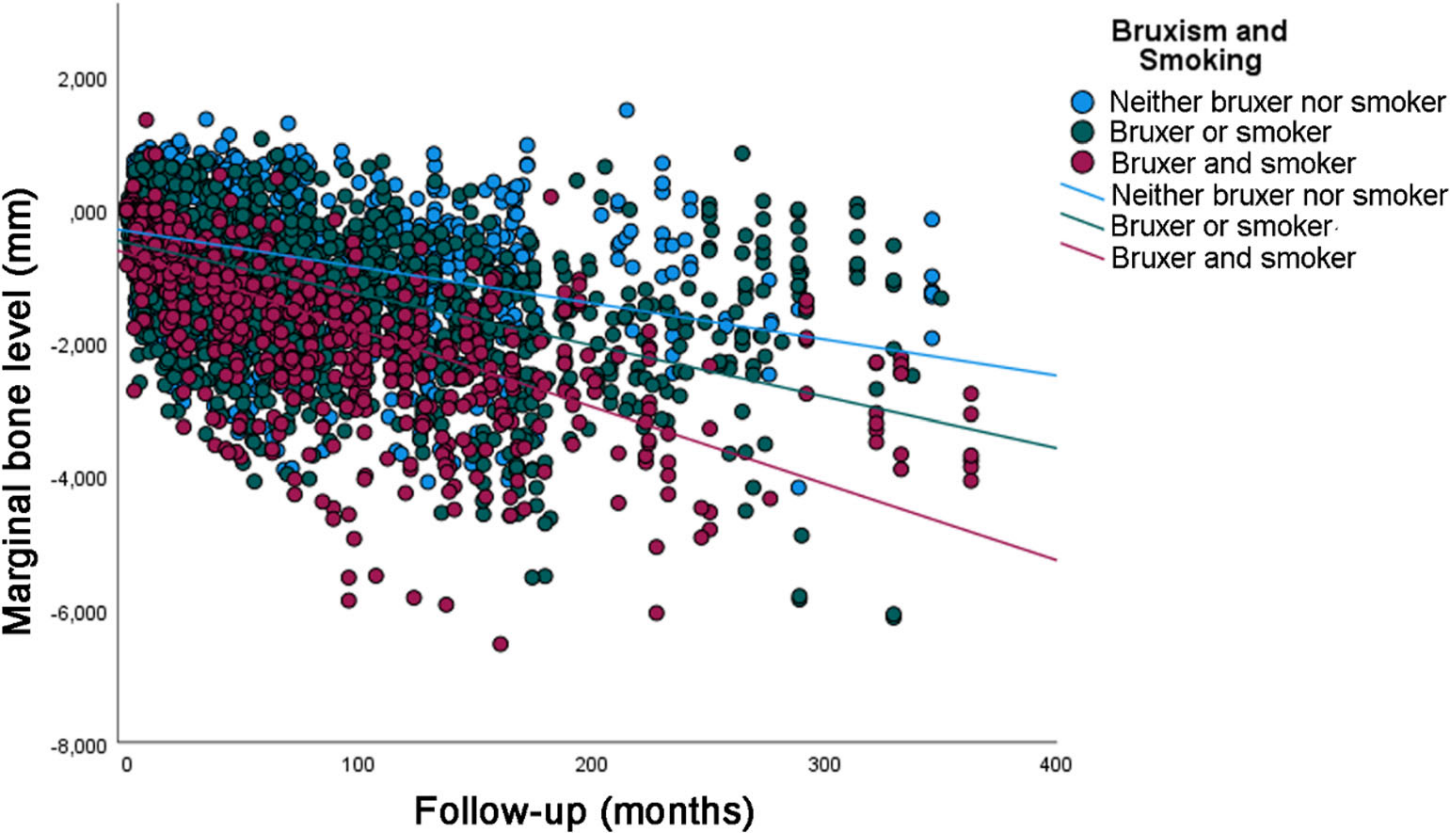
Scatter plot comparing the marginal bone level over time between groups of smokers, bruxers, neither, or both (linear regression).

An additional analysis was performed, investigating the possible synergistic effect of smoking and bruxism on MBL over time (Figure 4). It was observed that a greater MBL is observed when individuals are both bruxers and smokers (*y* = −0.65 − 0.01000*x*; *R* ^2^ linear = 0.459) when compared to individuals who are either bruxer or smoker (*y* = −0.49 − 0.00772*x*; *R* ^2^ linear = 0.280), or neither of them (*y* = −0.31 − 0.00545*x*; *R* ^2^ linear = 0.126), being statistically significant different (*p* < 0.001).

The results of the linear mixed‐effects model (Table 3) suggested that bruxism, smoking, age, region of the jaws, implant diameter, and prosthesis type had a statistically significant influence on MBL over time.

**TABLE 3:** Linear mixed‐effects model for MBL over time.

Predictor variables	*F* statistic	*p* value
Bruxism	469.117	**<0.001**
Smoking	58.896	**<0.001**
Age	6.743	**0.009**
Sex	2.884	0.090
Jaw	3.313	0.076
Region	62.439	**<0.001**
Implant surface	3.753	0.054
Implant diameter	4.195	**0.041**
Prosthesis type	13.043	**<0.001**
Prosthesis fixation	0.250	0.617

## 4 DISCUSSION

The aim of this study was to investigate whether bruxism leads to greater MBL around dental implants in comparison to non‐bruxers. According to the present results, bruxers presented a statistically significant greater MBL over time than non‐bruxers, especially so when also being a smoker. Therefore, the null hypothesis was rejected.

These results add evidence to the theory stating that bruxism could be a risk factor for MBL surrounding dental implants.^32^ The reasoning behind this theory is twofold: (1) Studies have found that bruxers have an increased bite force,^33^ which in combination with the lack of periodontal receptors controlling force application surrounding implants^34^ might lead to a greater risk of overloading; and (2) The grinding movements associated with bruxing episodes are more likely to expose implants to unfavorable non‐axial forces leading to high‐stress values on the surrounding bone.^32^ A finite element analysis study on the effect of different loading conditions on implants showed that the resulting strain intensity in the bone is highest around the cervical neck of the implant. The same study also showed that the stress on the cervical bone increases with more horizontal forces.^35^ The overloading combined with high values of eccentric stress is thought to cause microfractures in the surrounding bone crest and disturb the natural balance between bone‐remodeling and resorption, leading to greater MBL in bruxers when compared to non‐bruxers.^36^, ^37^ As far as the authors of the present study are aware, this is the first human clinical study to compare MBL around implants between balanced groups of bruxer and non‐bruxer patients. Therefore, there are no previous studies to which the present MBL results can be compared to, at least when it comes to bruxism.

Smoking was also suggested to have a significant effect on MBL over time. This is in agreement with the results of previous reviews on the subject, which have found smoking to be a risk factor for both implant failure and increased MBL around implants.^38^, ^39^ Thus, higher MBL around implants in smokers in comparison to non‐smokers has been observed in a series of clinical studies.^38^ Exposure to nicotine is believed to be the mechanism behind this correlation, as it causes vasoconstriction, reducing blood flow and the supply of nutrients necessary for bone formation and remodeling.^40^ Moreover, nicotine has been shown to suppress gene expression of certain enzymes involved in the regulation of osteoblast differentiation, proliferation, and apoptosis, which directly affects the bone remodeling process.^41^

A synergetic effect of bruxism and smoking was suggested by the present study. Patients who were both bruxers and smokers showed significantly more MBL over time in comparison to patients who were either a smoker or bruxer. This effect is maybe not so surprising when taking into consideration that both smoking and bruxism have a negative effect on MBL. The effects of smoking, that is, the reduced supply of nutrients^40^ together with the detrimental effects on osteoblasts,^41^ most likely hinder the body's ability to adapt to the overload caused by bruxism, hypothetically leading to greater MBL.

The region of implant installation was found to be a significant factor regarding MBL, with implants in the molar and incisor regions showing greater bone loss. This might be due to the fact that implants placed posteriorly in the jaws are more exposed to unfavorable forces during both chewing and bruxism. The occlusal loads are three times higher in the posterior regions compared to anterior regions.^42^ Anteriorly, the cause could be of a traumatic type, as the anterior maxilla is an area that is frequently exposed to trauma.^43^, ^44^ Another reason may be the eccentric stress caused by the axis of loading on implants in the anterior maxillary region.^43^ Higher values of MBL in the anterior maxilla in relation to the other regions have been observed in a clinical study.^45^ However, some studies have observed no difference in MBL between anterior and posterior regions of the jaws.^46^, ^47^

MBL around implants also varied depending on the type of prosthesis the implants were supporting. Overdentures represented more bone loss over time compared to other prosthetic constructions such as crowns or FDP. However, as only seven patients with overdentures were included in this study, the small sample size may lead to a high margin of error. The study also included twice as many bruxers with overdentures as non‐bruxers, which naturally leads to a non‐representative result.

There was a significant relationship between age and MBL over time, with increased bone loss in older patients. This corresponds with the knowledge that bone turnover naturally decreases with age, which may lead to an increased loss of bone mass surrounding both teeth and implants.^48^, ^49^ Low local bone density in the jaws has been associated with advanced age (>50 years).^50^ A positive correlation between patient age and MBL was also observed in other clinical studies,^45^, ^51^ although there are contrasting results in the literature.^47^

Other factors that showed a correlation with increased MBL were implant diameter, region of implant, and type of prosthesis, but the categories of these variables were very unbalanced in frequency, making it impossible to draw robust conclusions from these factors.

Although the patients suspected to be bruxers were called back for clinical examination in order to be able to classify them as probable bruxers, most of the study had a retrospective nature, meaning that all the used data were extracted from patient records. Retrospective studies, while cost‐ and time‐efficient, have several limitations due to their design. Because they are based on review of patient records that were not specifically written with the purpose of collecting data for research, there is always a risk that potentially important information was not recorded,^52^ such as the fabrication and frequency of use of night guards by the patients. The present results may however be applied to the general population, as there were no restrictions concerning the inclusion of patients other than the radiological follow‐up.

There is a need for further research into this topic in order to add more evidence to the suggestion that bruxism can be considered a risk factor for implant treatment, although not a contraindication.^16^

In conclusion, the present results suggest a significant correlation between bruxism and increased MBL over time around dental implants. In addition, it is suggested that older age, smoking, as well as the combination of bruxism and smoking further increase the risk of MBL over time.

## AUTHOR CONTRIBUTIONS

Camila Vu: Investigation, Writing‐original draft, Writing‐review & editing.

Clara Bredberg: Investigation, Writing‐original draft, Writing‐review & editing.

Birgitta Häggman‐Henrikson: Conceptualization, Writing‐review & editing, Visualization, Supervision.

Bruno R. Chrcanovic: Conceptualization, Methodology, Investigation, Data curation, Formal analysis, Writing‐original draft, Writing‐review & editing, Visualization, Supervision.

## CONFLICT OF INTEREST

The authors declare no conflict of interest.

## Data Availability

The data that support the findings of this study are available on request from the corresponding author. The data are not publicly available due to privacy or ethical restrictions.
